# Monitoring childbirth care in primary health facilities: a validity study in Gombe State, northeastern Nigeria

**DOI:** 10.7189/jogh.09.020411

**Published:** 2019-12

**Authors:** Antoinette Alas Bhattacharya, Elizabeth Allen, Nasir Umar, Adamu Umar Usman, Habila Felix, Ahmed Audu, Joanna RM Schellenberg, Tanya Marchant

**Affiliations:** 1 Department of Disease Control, London School of Hygiene & Tropical Medicine, London, UK; 2 Department of Medical Statistics, London School of Hygiene & Tropical Medicine, London, UK; 3 Data Research and Mapping Consult, Abuja, Nigeria; 4 State Primary Health Care Development Agency, Gombe, Nigeria

## Abstract

**Background:**

Improving the quality of facility-based births is a critical strategy for reducing the high burden of maternal and neonatal mortality and morbidity across all settings. Accurate data on childbirth care is essential for monitoring progress. In northeastern Nigeria, we assessed the validity of childbirth care indicators in a rural primary health care context, as documented by health workers and reported by women at different recall periods.

**Methods:**

We compared birth observations (gold standard) to: (i) facility exit interviews with observed women; (ii) household follow-up interviews 9-22 months after childbirth; and (iii) health worker documentation in the maternity register. We calculated sensitivity, specificity, and area under the receiver operating curve (AUC) to determine individual-level reporting accuracy. We calculated the inflation factor (IF) to determine population-level validity.

**Results:**

Twenty-five childbirth care indicators were assessed to validate health worker documentation and women’s self-reports. During exit interviews, women’s recall had high validity (AUC≥0.70 and 0.75<IF<1.25) for 9 of 20 indicators assessed; six additional indicators met either AUC or IF criteria for validity. During follow-up interviews, women’s recall had high validity for one of 15 indicators assessed, placing the newborn skin-to-skin; two additional indicators met IF criteria only. Health worker documentation had high validity for four of 10 indicators assessed; three additional indicators met AUC or IF criteria.

**Conclusions:**

In addition to standard household surveys, monitoring of facility-based childbirth care should consider drawing from and linking multiple data sources, including routine health facility data and exit interviews with recently delivered women.

The childbirth process presents a time of great risk of death for women and their newborns [1,2]. Of the estimated 303 000 maternal deaths and 2.5 million neonatal deaths that occurred in 2015, 113 000 maternal deaths and over 1 million neonatal deaths were attributed to complications from childbirth and the immediate postpartum period [3,4]. The distribution of this risk of death is uneven. While 36% of the world’s population lives in sub-Saharan Africa and Southern Asia, these regions account for 86% of maternal deaths and at least 78% of the newborn deaths [1-5]. For facility-based births, improving the quality of care for women and newborns especially during the intrapartum period is considered one of the most effective strategies for reducing maternal and neonatal mortality and morbidity across all settings [1,6-10].

Global and national monitoring of facility-based care often includes self-reported retrospective data collected in household surveys such as the Demographic and Health Survey (DHS) and Multiple Indicator Cluster Survey (MICS) [11-13]. For population-based coverage estimates of childbirth care, these periodic and nationally representative surveys collect a limited set of data which include maternal background characteristics and birth history, delivery by a skilled birth attendant, and newborn care practices [14,15]. A small number of criterion validity studies of childbirth care which measured the extent to which the women’s self-reported data at different recall periods align with a gold standard, have demonstrated mixed results on the accuracy of data in household surveys [16-22]. Understanding how best to accurately monitor childbirth care is an emerging research priority and evidence from different contexts is required [23,24].

Routine data can be used to monitor the content of facility-based care, but concerns about completeness, consistency, and accuracy have hampered their use [13]. Most studies on the accuracy of routine data have focused on verifying the aggregate data reported by facilities to higher management levels and comparing these to data documented by health workers [25-30]. However, similar to the population-based surveys, the extent to which the data documented by health workers reflect the “truth” of care is also not well-established [31].

In the high mortality setting of northeastern Nigeria, we assessed the extent to which different data recording methods could contribute to the global- and national-level monitoring of maternal and newborn health. Using direct birth observations as a gold standard, we compared these observations to: (i) facility exit interviews with women after childbirth; (ii) household follow-up interviews with women nine to 22 months after childbirth; and (iii) health worker documentation of childbirth events in the facility maternity register.

## METHODS

### Ethical review

Study approvals were obtained from the London School of Hygiene & Tropical Medicine (reference 14091) and the Health Research Ethics Committees for Nigeria (reference NHREC/01/01/2007) and Gombe State (reference ADM/S/658/Vol. II/66).

### Study setting

Gombe State, northeastern Nigeria, has high maternal and newborn mortality at 814 per 100 000 live births and 35 per 1000 live births, respectively; nationally, maternal mortality estimates are also 814 per 100 000 live births and neonatal mortality estimates are 39 per 1000 live births [3,4,14,15]. Gombe is predominantly rural and 44% of the population have some primary school education. Most women access maternity care through public facilities. Seventy-two percent of women reported at least one antenatal care visit during their last pregnancy and 29% gave birth in a health facility [15]. In 2018, over 70% of facility deliveries took place in rural primary health facilities [32].

### Indicator selection

Twenty-five indicators were selected, focusing on the content of childbirth care (**Table 1**): skilled birth attendance and companionship during labor and delivery; care for the woman (maternal background characteristics, provider practices and respectful care, clinical care); and care for the newborn (immediate postnatal care and newborn outcomes). To select these indicators, we referred to the Ending Preventable Maternal Mortality and Every Newborn Action Plan strategy documents for priority indicators to monitor progress towards Sustainable Development Goals targets [33,34]. We also sought to complement indicators collected in the Nigeria Demographic and Health Survey as well as earlier studies validating childbirth care indicators [14,16-20].

**Table 1:** Childbirth care indicators and data recording methods compared with birth observations (gold standard) for validation analyses

		Comparison data recording method*		
**Indicator**	**Births observation**	**Facility exit interview**	**Household follow-up interview**	**Facility maternity register**
**Skilled birth attendance and companionship during labor and delivery:**				
Main provider – doctor, nurse, or midwife	X	X	X	X
More than one provider present at birth	X	X	X	
Support person present at birth	X	X	X	
**Care for the woman:**				
Maternal background†:				
Age at delivery (adolescent births)	X			X
Prior parity (prior parity, 4 or more births)	X			X
Provider practices and respectful care:				
Woman allowed to move and change position during labor	X	X		
Woman allowed to drink liquids and eat during labor	X	X		
Woman allowed to deliver in preferred position	X	X		
Woman allowed to have a support person at birth	X	X	X	
Birth attendant washed hands with soap before examinations	X	X	X	
Birth attendant wore gloves during examinations	X	X	X	
Partograph used to monitor labor and delivery	X			X
Clinical care:				
Blood pressure taken – initial client assessment	X	X	X	
Episiotomy performed	X	X	X	
Prophylactic uterotonic administered during third stage of labor to prevent postpartum hemorrhage	X	X	X	X
**Care for the newborn:**				
Immediate postnatal care:				
Mother and newborn kept in same room after delivery	X	X	X	
Newborn immediately dried with a towel	X	X	X	
Newborn immediately placed skin-to-skin	X	X	X	
Immediate initiation of breastfeeding	X	X	X	
Essential newborn care‡	X	X	X	X
Chlorhexidine applied to newborn's cord to prevent infection	X	X		
Baby weighed at birth	X	X	X	X
Newborn outcomes:				
Low birthweight, <2500 g†	X	X		X
Pre-term birth	X			X
Stillbirth, fresh or macerated	X			X
Total indicators	25	20	15	10

*Observed women were interviewed before discharge from the facility (exit interview) and at home nine to 22 mo after childbirth (follow-up interview). Health workers documented childbirth events in facility maternity registers.

**†**For validation analyses, the following indicators were converted into binary variables: age at delivery (adolescent births); prior parity (prior parity, four or more births); and baby’s birthweight (low birthweight, <2500 g).

**‡**In the facility maternity register, essential newborn care is a composite indicator for (i) immediate initiation of breastfeeding and (ii) baby kept warm.

In Gombe, maternity registers defined essential newborn care as the immediate initiation of breastfeeding and the baby being kept warm within 30 minutes of birth [35]. To determine if the maternity register provided a sufficient approximation to globally-defined indicators, we compared the maternity register’s essential newborn care data to being kept warm and the initiation of breastfeeding within the first hour of birth [34]. For validation analyses, the following indicators were converted into binary variables: maternal age at delivery (adolescent births); prior parity (prior parity, four or more births); and baby’s birthweight (low birthweight, <2500 g).

### Study sites and data sources

**Figure 1:**
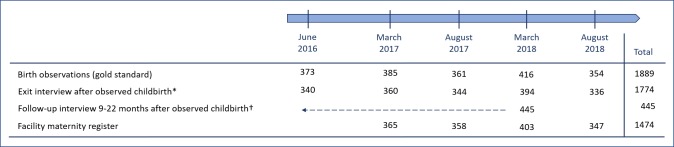
Data recording methods, data collection rounds, and the number of women observed and interviewed. *Of the 1889 women observed, 115 (6%) did not participate in an exit interview: 11 (0.5%) were discharged with their newborn and refused to be interviewed; 104 (5.5%) women were not interviewed (21 were referred to another facility; 61 still births with 1 maternal death; 22 newborn deaths). †A total of 445 women were followed up at home in March 2018, 9-22 months after their observed childbirth: 147 women from deliveries in June 2016; 146 women from deliveries in March 2017; 152 women from deliveries in August 2017.

As part of an initiative to improve care in Gombe State, data were collected between 2016-2018, including facility-based birth observations [36]. A summary of each data recording method is provided in **Figure 1**; detailed descriptions follow.

#### Birth observations

Starting in June 2016, five rounds of birth observations took place in 10 primary health facilities. Each round took place roughly every six months and lasted three weeks. To select the facilities for birth observations, a state-wide random sample of 107 facilities was drawn in November 2015 from approximately 500 government-owned primary health facilities. The maternity registers were reviewed to determine the volume of births occurring in the previous six months. The 10 facilities with the highest number of births were selected for birth observations [37]. An average of 15.7 births (standard deviation SD = 12.0) occurred per month in the 10 primary health facilities, compared to the state-level average of 4.3 births (SD = 6.3) per month in primary health facilities [38].

All women attending the facility for delivery were invited to participate, excluding women admitted for monitoring before the onset of labor. Women were given a description of the study and the procedures, including the right to withdraw participation at any time. A trained observer (local midwives, not employees of the assigned facility) stayed in the same room to continuously document labor and delivery processes through the first hour after birth, using a structured checklist. Labor and delivery took place in the same room. The mother and newborn were usually kept together until discharged from the facility.

Two observers and one clinical supervisor were assigned per facility to work in shifts and cover all deliveries. Although observers were trained midwives, they had no legal right to intervene in clinical care during the observation period because they were not employed in the same facilities where they were doing the observations. At all times during the observation, the observer prioritized safety of the mother and newborn over data collection; protocols were established on how to seek help in the event of any life-threatening event. Priorities for the supervisor were (i) to ensure that consenting procedures were carried out; (ii) to observe data collection and carry out interrater reliability checks; (iii) to assist in the case of a query from facility employees or from clients and families; (iv) to collect and check digital data at the end of each day.

Before each round, observers underwent four days of practical training to conduct unobtrusive observations, train on safety and confidentiality protocols, and ensure consistency of rating between observers. Observations were recorded onto a Lenovo A3300 tablet using CSPro version 7.0 (United States Census Bureau and ICF Macro, Suitland, MD, USA). Each observed woman was assigned a unique observation number to facilitate linking information to other data sets.

#### Facility maternity registers

Following the birth observation, regardless of newborn outcome, the observer extracted data about the woman from the maternity register. Data extraction took place on the same day as the observed birth after the first hour of birth. Data were directly entered into the tablet.

#### Facility exit interviews

Women were usually discharged within 24 hours of delivery. Each observed woman leaving the facility with a live newborn was invited to participate in an exit interview. The exit interview covered information recorded during the observation and harmonized with questions asked in the DHS and MICS. Each interview was conducted in Hausa by a member of the observation team assigned to the facility. Interview questions are available in Table S1 in **Online Supplementary Document****.**

#### Household follow-up interviews, nine to 22 months after childbirth

In addition to recall during exit interviews, it was of interest to understand the validity of women’s recall in the context of household surveys, such as DHS and MICS. For this purpose, we conducted household-level follow-up interviews with a subset of the observed women to recall childbirth events. To represent a range of recall periods that may be encountered during a household survey, in March 2018 we selected approximately 150 women from each of the first three rounds of birth observations which occurred in June 2016 (22 months recall), March 2017 (15 months recall), and August 2017 (9 months recall); this selection was done by a simple random sample of a de-identified list of women observed per round. Each interview was conducted in Hausa and the women were asked the same questions as in the exit interview.

### Sample size

To estimate the sample size, 50% prevalence from clinical observations (gold standard) was set for all indicators as we expected variability in the frequency of indicators. Sensitivity was set at 60% ± 7% precision and specificity at 70% ± 7% precision. Type 1 error was set at 0.05, assuming a normal approximation to a binomial distribution. Thus, a minimum sample size of 400 was required for observed women at exit interviews, at follow-up interviews, and in the maternity register.

### Analysis

To combine the data from five rounds of data collection, we tested for marginal homogeneity using Yang’s chi-square test for clustered binary matched pair data using the clust.bin.pair package in R [39,40]. Of the 45 matched pairs analyzed (see **Table 1**), one indicator showed evidence of clustering across time when comparing birth observations and women’s self-reports at exit and follow-up interviews: birth attendant washed hands with soap before examinations. Given the number of matched pairs analyzed, we considered there to be sufficient evidence that the data collection rounds could be combined.

Validation analyses were performed using Stata 14.2 (Stata Corp, College Station, TX, USA) [41]. Using birth observations as the gold standard, we assessed each indicator’s validity at the individual- and population-level.

To measure individual-level reporting accuracy, we constructed three two-by-two tables for each indicator which compared the birth observation to each data recording method [16,18-20,23]. Missing and “don’t know” responses were excluded from the two-by-two tables. We calculated percent agreement between the birth observation and each data recording method.

For two-by-two tables with at least five observations per cell, we calculated the sensitivity (true positive rate) and specificity (true negative rate) for each indicator. We quantified the area under the receiver operating characteristic curve (AUC) and estimated 95% confidence intervals (CI) assuming a binomial distribution. AUC values range from 0 to 1, with 0.5 representing a random guess and 1 representing complete accuracy. An AUC value of 0.7 or higher was chosen as the cutoff criteria for high individual-level reporting accuracy [23].

To measure the population-level validity, we calculated each indicator’s inflation factor (IF), which is the ratio of the estimated population-based survey prevalence to the gold standard’s prevalence. The IF reflects the degree to which an indicator would be over- or under-estimated in a population-based survey. To estimate the population-based survey prevalence, we used the following equation [42]: estimated population survey prevalence = (gold standard prevalence × sensitivity) + [(1 – gold standard prevalence) × (1 – specificity)]. An IF value between 0.75 and 1.25 was the chosen cut-off criteria for low population-level bias [23].

## RESULTS

### Sample description

Characteristics of the women observed during childbirth are presented in **Table 2**. Women’s age ranged from 15 to 47 years, with a median age of 24 years (interquartile range (IQR) = 20-28). Forty-four percent of women had at least 4 prior deliveries, 47% of women had no formal education, and 99% were married.

**Table 2:** Characteristics of women observed during childbirth

	Number of women, N(%), N = 1774*
**Data collection round:**	
June 2016	340 (19)
March 2017	360 (20)
August 2017	344 (19)
March 2018	394 (22)
August 2018	336 (19)
**Age of client at delivery:†**	
15-19	351 (20)
20-24	600 (34)
25-29	402 (23)
30-34	243 (14)
35-39	126 (7)
40+	47 (3)
**Prior parity:‡**	
0	41 (2)
1	355 (20)
2	339 (19)
3	255 (14)
4 or more	779 (44)
**Educational attainment:**	
None	827 (47)
Primary	412 (23)
Secondary	490 (28)
Higher	45 (3)
**Marital status:**	
Single, never married	12 (1)
Married	1759 (99)
Widowed	3 (0)
**Time of delivery:§**	
Day, 8:00am-6:59pm	1038 (59)
Night, 7:00pm-7:59am	715 (40)
**Day of delivery:‖**	
Weekday	1194 (67)
Weekend	567 (32)
**Main provider during labor and delivery:¶**	
Doctor, nurse, or midwife	184 (10)
Community health extension worker, junior CHEW	690 (39)
Hospital assistant	387 (22)
Other facility staff	461 (26)
Other non-staff, including traditional birth attendant	51 (3)

CHEW – community health extension worker

*Distribution of characteristics based on the 1774 respondents during exit interviews. Percentages do not always add up to 100% due to rounding, missing responses (up to 1.1%), and “don’t know” responses (0.2%).

†“Age of client at delivery” had 1 (0.1%) missing response and 4 (0.2%) “don’t know” responses.

‡“Prior parity” had 6 (0.3%) missing responses.

§“Time of delivery” had 19 (1.1%) missing responses.

‖“Day of delivery” had 13 (0.7%) missing responses.

¶“Main provider during labor and delivery” had 1 (0.1%) missing response.

**Figure 2:**
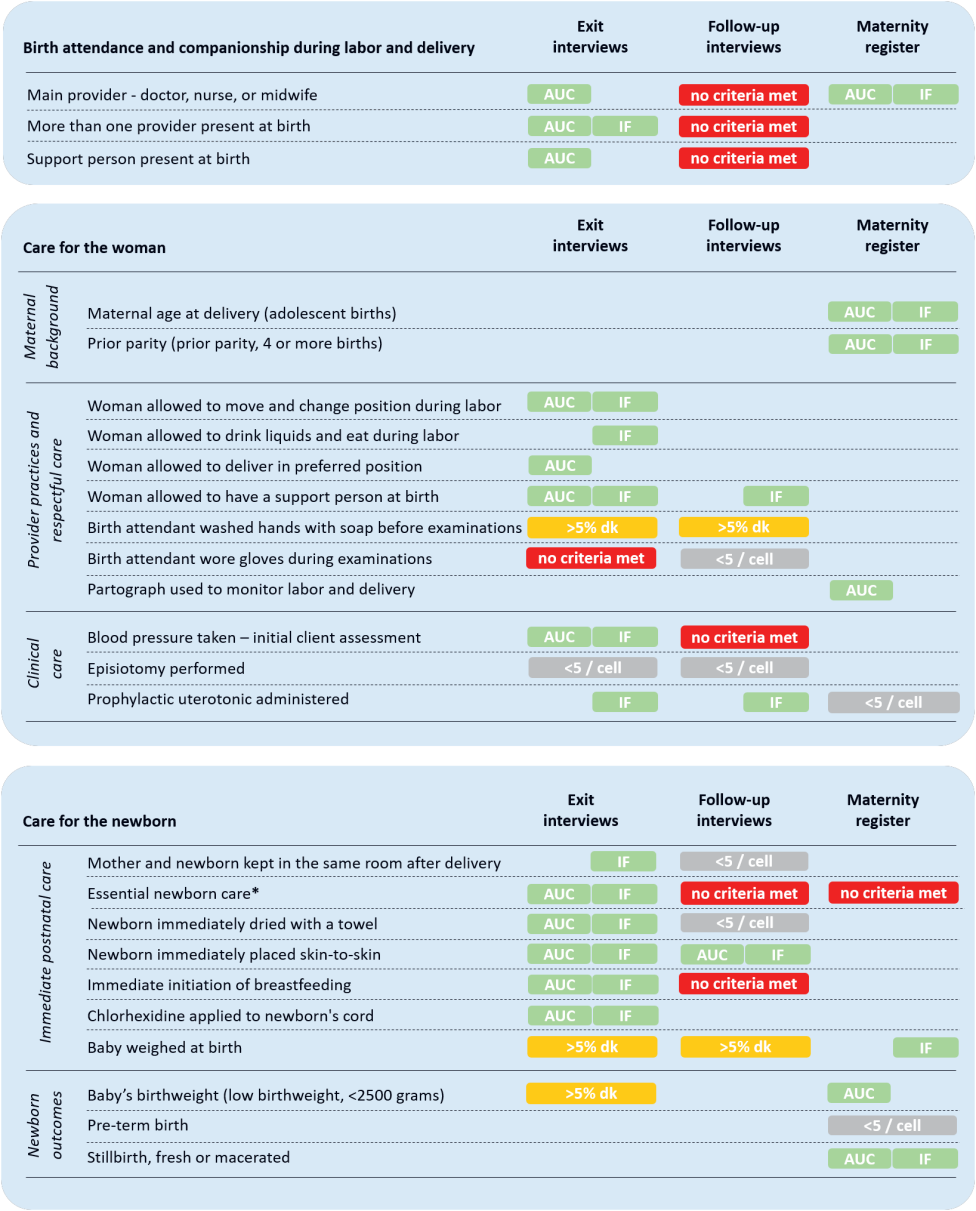
Summary of childbirth care indicator validity criteria across data recording methods. Observed women were interviewed before discharge from the facility (exit interview) and at home nine to 22 months after childbirth (follow-up interview). Health workers documented childbirth events in facility maternity registers. AUC = area under the receiver operating characteristic curve; IF = inflation factor; >5%dk = >5% “don’t know” responses; <5/cell = less than 5 observations per cell in two-by-two table validating data recording method against gold standard; AUC criteria for high individual-level reporting accuracy: AUC≥0.7; IF criteria for low population-level bias: 0.75<IF<1.25. *In the facility maternity register, essential newborn care is a composite indicator for (i) immediate initiation of breastfeeding and (ii) baby kept warm.

For each indicator and data recording method: indicator prevalence, “don’t know” responses, percent agreement with gold standard, sensitivity, specificity, AUC, and IF values are summarized in **Table 3****.** **Figure 2** presents a summary of the validity criteria met across data recording methods.

**Table 3:** Validation analyses: comparing birth observations with women’s self-reports at facility exit interviews, women’s self-reports at household follow-up interviews nine to 22 months after childbirth, and health worker documentation in maternity registers

Birth observations (gold standard)		Comparison data recording method*,†				Matched pairs							
**N**	**Prevalence (95% CI)**		**N**	**Don’t know (%)**	**Prevalence (95% CI)**	**Matched pairs, N**	**Agreement (%)**	**5 counts per cell?**	**Sensitivity (95% CI)**	**Specificity (95% CI)**	**AUC‡ (95% CI)**	**IF‡**	**Criteria met‡**
**Skilled birth attendance and companionship during labor and delivery:**													
Main provider – doctor, nurse, or midwife:													
1889	10 (4-24)	Exit interview, women’s self-report	1775	0	32 (24-41)	1775	72	Yes	67 (60-74)	72 (70-75)	0.70 (0.66-0.73)	3.04	AUC
445	13 (5-27)	Follow-up interview, women’s self-report	426	0	48 (37-60)	426	55	Yes	62 (48-75)	54 (49-59)	0.58 (0.51-0.65)	3.76	none
1516	12 (4-31)	Maternity register, health worker documentation	1327	0	12 (4-29)	1327	97	Yes	92 (87-96)	98 (97-99)	0.95 (0.93-0.97)	1.05	AUC, IF
More than one provider present at birth:													
1869	57 (45-68)	Exit interview, women’s self-report	1774	0	58 (48-66)	1755	93	Yes	95 (93-96)	90 (88-92)	0.93 (0.91-0.94)	1.02	AUC, IF
444	55 (43-67)	Follow-up interview, women’s self-report	426	0	80 (67-89)	418	60	Yes	86 (80-90)	26 (20-33)	0.56 (0.52-0.60)	1.45	none
Support person present at birth:													
1127	35 (22-49)	Exit interview, women’s self-report	1075	0	57 (41-72)	1067	75	Yes	97 (94-98)	64 (60-67)	0.80 (0.78-0.82)	1.65	AUC
443	31 (17-50)	Follow-up interview, women’s self-report	426	0	82 (74-88)	419	41	Yes	88 (81-93)	21 (16-26)	0.54 (0.51-0.58)	2.64	none
**Care for the woman:**													
Maternal age at delivery (adolescent births):**§**													
1516	20 (17-24)	Maternity register, Health worker documentation	1472	0	20 (17-23)	1463	98	Yes	95 (92-97)	99 (98-100)	0.97 (0.96-0.98)	0.98	AUC, IF
Prior parity (prior parity, 4 or more births):**§**													
1515	47 (42-52)	Maternity register, health worker documentation	1474	0	49 (43-56)	1471	93	Yes	95 (94-97)	91 (88-93)	0.93 (0.92-0.94)	1.06	AUC, IF
Woman allowed to move and change position during labor:													
712	78 (66-87)	Exit interview, women’s self-report	1075	1	71 (58-81)	674	84	Yes	92 (89-94)	57 (49-66)	0.75 (0.70-0.79)	1.04	AUC, IF
Woman allowed to drink liquids and eat during labor:													
712	91 (88-93)	Exit interview, women’s self-report	1075	1	92 (88-94)	674	91	Yes	98 (96-99)	25 (14-37)	0.61 (0.56-0.67)	1.05	IF
Woman allowed to deliver in preferred position:													
773	52 (43-61)	Exit interview, women’s self-report	1075	1	75 (66-83)	691	81	Yes	95 (92-97)	63 (58-69)	0.79 (0.76-0.82)	1.29	AUC
Woman allowed to have support person at birth:													
1885	63 (43-80)	Exit interview, women’s self-report	1773	1	59 (41-75)	1757	85	Yes	85 (83-87)	85 (82-88)	0.85 (0.83-0.87)	0.94	AUC, IF
443	60 (36-80)	Follow-up interview, women’s self-report	426	1	67 (56-77)	422	57	Yes	71 (65-76)	36 (29-44)	0.53 (0.49-0.58)	1.14	IF
Birth attendant washed hands with soap before examinations:													
1872	30 (21-41)	Exit interview, women’s self-report	1775	16	40 (31-49)	1492	81	Yes**¶**	–	–	–	–	–
444	19 (13-26)	Follow-up interview, women’s self-report	426	16	77 (65-85)	359	26	No	–	–	–	–	–
Birth attendant wore gloves during examinations:													
1872	75 (66-83)	Exit interview, women’s self-report	1775	0	98 (98-99)	1769	75	Yes	98 (98-99)	2 (1-3)	0.50 (0.49-0.51)	1.30	none
444	90 (73-97)	Follow-up interview, women’s self-report	426	1	98 (94-99)	419	90	No	–	–	–	–	–
Partograph used to monitor labor and delivery:													
1516	21 (14-29)	Maternity register, health worker documentation	1308	0	39 (29-50)	1306	79	Yes	95 (91-97)	75 (72-77)	0.84 (0.83-0.87)	1.92	AUC
Blood pressure taken – initial client assessment:													
1515	31 (21-44)	Exit interview, women’s self-report	1435	0	34 (23-47)	1429	92	Yes	91 (88-94)	92 (90-93)	0.91 (0.90-0.93)	1.10	AUC, IF
444	22 (10-43)	Follow-up interview, women’s self-report	426	2	58 (48-67)	417	56	Yes	82 (73-89)	48 (43-54)	0.65 (0.60-0.70)	2.62	none
Episiotomy performed:													
1504	1 (1-2)	Exit interview, women’s self-report	1435	0	2 (1-3)	1429	98	No	–	–	–	–	–
444	1 (0-4)	Follow-up interview, women’s self-report	426	2	9 (1-17)	424	91	No	–	–	–	–	–
Prophylactic uterotonic administered during third stage of labor to prevent postpartum haemorrhage:													
1867	96 (93-98)	Exit interview, women’s self-report	1775	0	94 (92-95)	1763	93	Yes	95 (94-96)	33 (22-45)	0.64 (0.58-0.70)	0.98	IF
442	96 (93-97)	Follow-up interview, women’s self-report	426	1	83 (78-87)	420	82	Yes	84 (80-88)	26 (9-51)	0.55 (0.45-0.66)	0.88	IF
1501	96 (92-98)	Maternity register, health worker documentation	1338	0	93 (90-95)	1332	90	No	–	–	–	–	–
**Care for the newborn:**													
Mother and newborn kept in the same room after delivery:													
1755	97 (96-98)	Exit interview, women’s self-report	1775	1	96 (94-97)	1704	97	Yes	98 (97-99)	40 (24-58)	0.69 (0.61-0.77)	1.00	IF
427	95 (90-98)	Follow-up interview, women’s self-report	426	0	88 (81-93)	406	85	No	–	–	–	–	–
Essential newborn care:**‖**													
1889	42 (29-56)	Exit interview, women’s self-report	1774	0	51 (36-65)	1774	87	Yes	92 (90-94)	83 (81-85)	0.88 (0.86-0.89)	1.15	AUC, IF
445	36 (19-57)	Follow-up interview, women’s self-report	445	0	64 (44-80)	445	60	Yes	83 (76-89)	47 (41-53)	0.65 (0.61-0.69)	1.77	none
1516	39 (26-53)	Maternity register, health worker documentation	1297	0	95 (90-97)	1297	44	Yes	97 (95-98)	6 (5-8)	0.52 (0.51-0.53)	2.46	none
Newborn immediately dried with a towel:													
1472	95 (89-98)	Exit interview, women’s self-report	1435	2	94 (89-97)	1393	98	Yes	99 (98-99)	68 (53-81)	0.83 (0.77-0.90)	1.00	AUC, IF
430	91 (81-96)	Follow-up interview, women’s self-report	426	3	91 (78-97)	397	86	No	–	–	–	–	–
Newborn immediately placed skin-to-skin:													
1759	77 (57-89)	Exit interview, women’s self-report	1775	3	72 (53-85)	1681	94	Yes	94 (92-95)	93 (90-95)	0.93 (0.92-0.95)	0.96	AUC, IF
427	67 (38-87)	Follow-up interview, women’s self-report	426	1	75 (50-90)	402	78	Yes	90 (86-94)	52 (43-61)	0.71 (0.66-0.76)	1.15	AUC, IF
Immediate initiation of breastfeeding:													
1744	49 (34-63)	Exit interview, women’s self-report	1775	2	51 (37-65)	1686	90	Yes	94 (92-95)	87 (84-89)	0.90 (0.89-0.92)	1.08	AUC, IF
424	42 (23-63)	Follow-up interview, women’s self-report	426	0	67 (46-83)	404	62	Yes	87 (81-92)	44 (38-50)	0.66 (0.62-0.70)	1.66	none
Chlorhexidine administered to newborn’s cord to prevent infection:													
1425	85 (74-92)	Exit interview, women’s self-report	1435	5	80 (68-89)	1323	95	Yes	96 (95-97)	84 (77-89)	0.90 (0.87-0.93)	0.99	AUC, IF
Baby weighed at birth:													
1785	84 (76-90)	Exit interview, women’s self-report	1774	9	80 (64-90)	1603	86	Yes**¶**	–	–	–	–	–
439	78 (71-84)	Follow-up interview, women’s self-report	426	11	62 (40-80)	377	66	Yes**¶**	–	–	–	–	–
1439	90 (76-96)	Maternity register, health worker documentation	1421	0	94 (84-98)	1403	87	Yes	95 (93-96)	16 (10-23)	0.55 (0.52-0.58)	1.04	IF
Newborn outcome: low birth weight**§**:													
1604	5 (3-8)	Exit interview, women’s self-report	1424	57	3 (1-5)	606	98	Yes**¶**	–	–	–	–	–
1432	6 (3-10)	Maternity register, health worker documentation	1338	0	17 (9-32)	1180	83	Yes	62 (50-73)	85 (83-87)	0.73 (0.68-0.79)	3.22	AUC
Newborn outcome: pre-term birth:													
1515	2 (1-7)	Maternity register, health worker documentation	1169	0	2 (1-7)	1167	99	No	–	–	–	–	–
Newborn outcome: stillbirth:													
1516	3 (1-6)	Maternity register, health worker documentation	1375	0	3 (1-5)	1365	99	Yes	82 (66-92)	100 (99-100)	0.91 (0.84-0.97)	0.97	AUC, IF

CI – confidence interval, IF – inflation factor, AUC - area under the receiver operating curve

*Observed women were interviewed before discharge from the facility (exit interview) and at home nine to 22 mo after childbirth (follow-up interview). Health workers documented childbirth events in facility maternity registers.

†For health worker documentation in facility maternity registers, data were available for 4 of 5 data collection rounds (N = 1516 observations): March 2017, August 2017, March 2018, and August 2018. For exit interviews, data from June 2016 data collection (round 1) were not available for: (i) blood pressure taken – initial client assessment; (ii) episiotomy performed; (iii) newborn immediately dried with a towel; and (iv) chlorhexidine applied to newborn’s cord to prevent infection. For exit interviews, data from June 2016 and March 2017 data collection (rounds 1 and 2) were not available for: (i) woman allowed to move and change positions during labor; (ii) woman allowed to drink and eat during labor; and (iii) woman allowed to deliver in preferred position.

‡AUC = area under the receiver operating characteristic curve; IF = inflation factor. AUC criteria for high reporting accuracy: AUC≥0.7; IF criteria for low population bias: 0.75<IF<1.25.

§For validation analyses, the following indicators were converted into binary variables: age at delivery (adolescent births); prior parity (prior parity, four or more births); and baby’s birthweight (low birthweight, <2500 g).

‖In the facility maternity register, essential newborn care is a composite indicator for (i) immediate initiation of breastfeeding and (ii) baby kept warm.

¶For these indicators with >5 observations per cell, validation analyses were not conducted due to >5% “don’t know” responses.

 “Don’t know” responses, which indicate the extent to which recall may or may not be possible, were greater than 5% for: birth attendant washed hands with soap before examinations (exit and follow-up); baby weighed at birth (exit and follow-up); and low birthweight (exit only). Health workers documented in maternity registers most frequently for: baby weighed at birth (99% completeness), maternal age at delivery (97%), and prior parity (97%). Documentation was least frequent for the composite indicator essential newborn care (82% completeness) and pre-term birth (77%).

### Skilled birth attendance and companionship during labor and delivery

Health worker documentation of the main provider’s cadre had high overall validity, meaning AUC≥0.70 for high individual-level accuracy and 0.75<IF<1.25 for low population-level bias. During exit interviews, women’s recall had high overall validity for the presence of more than one provider at birth and high individual-level accuracy for the main provider’s cadre and the presence of a support person during labor and delivery. During follow-up, women’s recall for these three indicators met neither validity criteria.

### Care for the woman

Health worker documentation in maternity registers had high overall validity for maternal age at delivery and prior parity and high individual-level accuracy for reporting the use of a partograph. While there was insufficient variation in responses for validation analysis, health worker documentation had near complete agreement with the gold standard for the administration of a prophylactic uterotonic.

During exit interviews, women’s recall on four provider respectful care indicators met at least one validity criteria, with high overall validity for two indicators: allowed to move and change positions during labor and allowed to have a support person during labor and delivery. During follow-up, women’s recall of being allowed to have a support person maintained low population-level bias only.

During exit interviews, women’s report of clinical care received had high overall validity for having her blood pressure taken before delivery and low population-level bias only for the administration of prophylactic uterotonic. During follow-up, only administration of a prophylactic uterotonic was able to maintain the low population-level bias.

### Care for the newborn

For two indicators requiring the mother’s involvement, immediate initiation of breastfeeding and placing the newborn skin-to-skin, women’s recall during exit interviews had high overall validity. During follow-up, women’s recall of her baby being placed skin-to-skin maintained high overall validity, whereas recall of immediate breastfeeding met neither validity criteria. Health worker documentation of these practices as a composite indicator of essential newborn care met neither validity criteria; health workers documented a 95% prevalence for being kept warm and initiation of breastfeeding within 30 minutes of birth whereas birth observations documented 39% prevalence for these practices within one hour of birth.

For additional immediate newborn care indicators assessed, women’s recall during exit interviews had high overall validity for immediate drying of the newborn and the application of chlorhexidine on the newborn’s cord. Women’s recall of whether she and her newborn were kept in the same room after delivery nearly met the criteria for high overall validity, AUC = 0.69 (95% confidence interval (CI) = 0.61-0.77) and IF = 1.00. For whether the baby was weighed at birth, health worker documentation met criteria for low population-level bias.

For indicators related to low prevalence newborn outcomes, health worker documentation met high overall validity for whether a baby was stillborn and high individual-level accuracy for whether a newborn had low birthweight.

## DISCUSSION

Providing high quality facility-based childbirth care with a skilled provider is essential for improving the health and survival of women and newborns. Accurate information on the care received is essential to monitoring progress. In Gombe state, where women predominantly seek childbirth care in rural primary health facilities, our study suggests that health worker documentation in facility registers, facility-level exit interviews, and household-level follow-up interviews can all contribute to accurate monitoring, but no individual method provided a broad understanding of the provision and experience of childbirth care.

Our validation of health worker documentation against a gold standard of birth observations differed from other accuracy studies of facility-based data. To date, studies assessed the extent to which data sources agreed when aggregated, reflecting the critical capacity to tally and report consistently between levels of the health system. Focusing on individual-level validity, health worker documentation had high validity (AUC≥0.70 and/or 0.75<IF<1.25) for select indicators about the main provider, maternal background characteristics, and newborn outcomes. Unsurprisingly, health workers were well-positioned to determine the provider’s cadre and newborn outcomes such as stillbirths. Maternal background characteristics were also relatively stable data which could be verified during the antenatal period.

However, health worker documentation did not meet any validity criteria for essential newborn care, a composite indicator of immediate breastfeeding and keeping the baby warm. As noted earlier, the prevalence for essential newborn care within 30 minutes of birth documented by the health worker was 95% (95% CI = 90%-97%), whereas the observed prevalence for immediate breastfeeding and placing the newborn skin-to-skin within one hour of birth was only 39% (95% CI = 26%-53%); health workers markedly overestimated the prevalence. Given the complexity of the essential newborn care definition, this may reflect the format of the documentation source which did not distinguish between care elements, as well as potential differences in interpretation between the observer and the health worker.

Our study adds new evidence to the validity of women’s self-reports at different recall periods and focused on women who delivered in rural primary health facilities. We found that exit interviews had high validity for four immediate newborn care practices: drying the newborn with a towel; placing the newborn skin-to-skin; immediate breastfeeding; and applying chlorhexidine to a newborn’s cord. In contrast to our study, two validation studies using hospital exit interviews in Mexico and Kenya did not report high validity for immediate drying of the newborn, placing the newborn skin-to-skin, and immediate breastfeeding [18,19]. Facility environment may explain part of the differences observed, which may in turn influence the frequency of “don’t know” responses or the low specificity from a positive facility reporting bias [18,19]. For example, in our study, the practice of placing the newborn with the mother immediately after birth was 97%, compared to 10% in Mexico and 58% in Kenya.

Similar to other validation studies, we found that women’s self-reports during follow-up nine to 22 months after childbirth had low validity across indicators assessed. Placing a newborn skin-to-skin immediately after birth was the one exception, consistent with a follow-up study in Mozambique which included a nation-wide sample of rural and urban health facilities, but inconsistent with the Kenyan study [16,20]. One possible explanation for this being a memorable event for northeastern Nigerian women may be that the practice of immediate skin-to-skin contrasts with longstanding cultural beliefs on early bathing of newborns and the negative perceptions of vernix [43,44].

Indicators that met criteria for low population-level bias only, such as the administration of prophylactic uterotonic (exit, follow-up), permission to drink and eat during labor (exit), and baby weighed at birth (maternity register) had high prevalence, which masked a high false positive rate among the small number of clients that did not receive the service. Thus, we recommend caution when interpreting these indicators and triangulation with other data sources.

Our findings highlight the importance of expanding the sources of data for monitoring the content of childbirth care. In addition to standard household surveys, monitoring of facility-based childbirth care should consider drawing from and linking multiple data sources including routine health facility data and exit interviews with recently delivered women. Facility-based routine data, such as registers, and exit interviews are useful sources for determining an accurate numerator when monitoring facility-based care; linkages to population-level data are still critical to determine the denominators for population in need and underserved subgroups [13]. At a global level, as greater emphasis is placed on respectful maternity care and the clients’ experience of care, exit interviews are being included in the monitoring frameworks for assessing the quality of facility-based care [45]. Further, recent calls for greater investment in routine health information systems, if successful, would allow for monitoring beyond the global- and national-levels, as routine data are available at a greater level of disaggregation and frequency [13,46,47].

The limitations of exit interviews and routine data still need careful consideration, however. Facility registers capture limited information about service delivery and, hence, provide a narrower but more frequent picture of quality of care. Health worker documentation and exit interviews are susceptible to reporting biases, whereby health workers record information only for the services they provide and women report receiving an intervention because of social desirability bias or a higher quality of care that might be assumed with a facility delivery [17,18].

Among the strengths of this study was the use of birth observations as the gold standard which was compared to facility exit interviews, household follow-up interviews, and health worker documentation in maternity registers. The longitudinal study design allowed us to assess the validity of women’s self-reports for different recall periods: before discharge from a facility and at nine to 22 months after childbirth, which more closely reflects the recall period and interviewing conditions of household surveys. Further, this study was novel as this setting was predominantly rural, based in the primary health care context, and included validation of health worker documentation in maternity registers. Among the limitations of the study, our findings primarily reflect the reporting accuracy of women who seek facility-based care. Further, women participating in household surveys are not usually interviewed twice; however, individual-level reporting accuracy decreased in our study which is different from what we would expect for repeated measurements. The gold standard could be susceptible to error from incorrect observer interpretation, errors in data recording, or changing behaviour because of the Hawthorne effect, even in the presence of quality control mechanisms [48]. Even with pre-testing, the questions in the exit and follow-up interviews may not have been interpreted as intended. Further, some observed indicators had such high or low coverage and were unsuitable for validation analyses. Finally, while not strictly a limitation, relatively stringent cut-off criteria were chosen for AUC and IF to align with other studies [23].

## CONCLUSION

The childbirth process presents a time of great risk of death for women and newborns. Health worker documentation, facility-level exit interviews, and household-level follow-up interviews with women after childbirth each have a role to play in the accurate monitoring of facility-based childbirth care to improve the health and survival of women and their newborns.

## Additional material

Online Supplementary Document
