# Supplementary material for: Monitoring childbirth care in primary health facilities: a validity study in Gombe State, northeastern Nigeria
Source: J Glob Health. 2019 Jul 25;9(2):020411. doi: 10.7189/jogh.09.020411 (PMC6657002; doi:10.7189/jogh.09.020411)
Supplement: Online Supplementary Document [file jogh-09-020411-s001.pdf]

J Glob Health 2019; 9: 020411

**Table S1. Childbirth care indicators assessed across data recording methods**

| Data recording method | Childbirth care indicator item/question |
|---|---|
| ***Birth attendance and companionship during labor and delivery*** | |
| *Main provider – doctor, nurse, or midwife* | |
| Facility exit interview, Household follow-up interview | Thinking about the main person helping you during birth, what was the cadre of that person? |
| Maternity register | Who took delivery of the child? |
| *More than one provider present at birth* | |
| Facility exit interview, Household follow-up interview | Did more than one health worker assist with the birth? |
| *Support person present at birth* | |
| Facility exit interview, Household follow-up interview | Did you have a support person present during labor and childbirth? |
| ***Care for the woman*** | |
| *Maternal age at delivery (adolescent births)* | |
| Maternity register | Age |
| *Prior parity (prior parity, four or more births)* | |
| Maternity register | Parity |
| *Woman allowed to move and change position during labor* | |
| Facility exit interview, Household follow-up interview | Were you encouraged to move and change position during labor? |
| *Woman allowed to drink liquids and eat during labor* | |
| Facility exit interview, Household follow-up interview | Were you encouraged to have some light food during labor and delivery? |
| *Woman allowed to deliver in preferred position* | |
| Facility exit interview, Household follow-up interview | Were you allowed to deliver in the position you wanted to deliver? |
| *Woman allowed to have a support person at birth* | |
| Facility exit interview, Household follow-up interview | Were you encouraged to have a support person present during labor and childbirth? |
| *Birth attendant washes hands with soap before examinations* | |
| Facility exit interview, Household follow-up interview | Did the birth attendant wash his/her hands with soap and water or use antiseptic before examining you? |
| *Birth attendant wears gloves during examinations* | |
| Facility exit interview, Household follow-up interview | Did the birth attendant wear gloves when examining you? |

| | |
|---|---|
| *Partograph used to monitor labor and delivery* | |
| Maternity register | Partograph used to monitor labor and delivery? [yes/no] |
| *Blood pressure taken – initial client assessment* | |
| Facility exit interview, Household follow-up interview | When you were there did anyone check your blood pressure (put a strap around your upper arm and take a measurement)? |
| *Episiotomy performed* | |
| Facility exit interview, Household follow-up interview | When you gave birth, did the attendant need to cut your privates to get the baby out (also called an episiotomy)? |
| *Prophylactic uterotonic administered during third stage of labor to prevent postpartum hemorrhage* | |
| Facility exit interview, Household follow-up interview | Immediately after the birth, were you given an injection or drugs to help stop the bleeding? (also called a uterotonic)? |
| Maternity register | Active management of third stage of labor? [yes/no] |
| **Care for the newborn** | |
| *Mother and baby kept in the same room after delivery* | |
| Facility exit interview, Household follow-up interview | Were you and the baby kept in the same room after delivery? |
| *Essential newborn care* | |
| Facility exit interview, Household follow-up interview | Immediately after the birth, did you put the baby to your breast to help start breastfeeding, with or without the help of the health worker? AND Immediately after the birth, was the baby placed on your body “skin to skin”? |
| Maternity register | Essential newborn care: immediate initiation of breastfeeding, baby kept warm |
| *Newborn immediately dried with a towel* | |
| Facility exit interview, Household follow-up interview | Immediately after the birth, was the baby dried with a towel or cloth? |
| *Newborn immediately placed skin-to-skin* | |
| Facility exit interview, Household follow-up interview | Immediately after the birth, was the baby placed on your body “skin to skin”? |
| *Immediate initiation of breastfeeding* | |
| Facility exit interview, Household follow-up interview | Immediately after the birth, did you put baby to your breast to help start breastfeeding, with or without the help of the health worker? |
| *Chlorhexidine applied to newborn's cord to prevent infection* | |
| Facility exit interview, Household follow-up interview | Did the health worker put chlorhexidine on the baby’s cord to prevent infection? |
| *Baby weighed at birth* | |

| | |
|---|---|
| Facility exit interview, Household follow-up interview | Was your baby weighed at birth? |
| Maternity register | <2500 grams or ≥2500 grams |
| *Baby's birthweight (Low birthweight, <2500 grams)* | |
| Facility exit interview, Household follow-up interview | If the baby was weighed, can you tell me the birthweight (kg)? |
| Maternity register | <2500 grams or ≥2500 grams |
| *Pre-term birth* | |
| Maternity register | Pre-term birth? [yes/no] |
| *Stillbirth, fresh or macerated* | |
| Maternity register | Stillbirth, fresh or macerated |
